# Outcomes and Feasibility of a 12-Week Physical Literacy Intervention for Children in an Afterschool Program

**DOI:** 10.3390/ijerph17093129

**Published:** 2020-04-30

**Authors:** Emily Bremer, Jeffrey D. Graham, John Cairney

**Affiliations:** 1 Faculty of Kinesiology and Physical Education, University of Toronto, Toronto, M5S 2W6, Canada; 2 INfant and Child Health (INCH) Lab, Department of Family Medicine, McMaster University, Hamilton, ON L8P 1H6, Canada; grahajd2@mcmaster.ca; 3 School of Human Movement and Nutrition Sciences, University of Queensland, St Lucia 4072, Australia; j.cairney@uq.edu.au

**Keywords:** physical literacy, movement competence, physical activity, school children

## Abstract

Children (*N* = 90) from eight afterschool programs (*n* = 4 experimental sites with 47 children; *n* = 4 control sites with 43 children), along with the program leaders (*N* = 7) from the experimental sites, participated in a 12-week physical literacy intervention. Children were assessed on their physical literacy (movement competence, affect, confidence, and motivation) pre- and post-intervention using a suite of assessment tools that included the PLAYfun assessment of movement competence and a comprehensive child questionnaire. Experimental participants engaged in a daily physical literacy intervention at their afterschool program; controls engaged in their usual afterschool programming. Experimental group program leaders were assessed on their cognitions pre- and post-training and post-intervention, alongside questions regarding program acceptability and feasibility. Program leaders’ perceived knowledge and self-efficacy to implement the physical literacy program increased (*p* < 0.05) from pre- to post-training and these effects were maintained at post-intervention. No group differences were observed in the change of children’s motor competence, self-efficacy, or motivation from baseline to post-intervention. However, significant increases in affect were observed among participants in the experimental group (*p* < 0.05). Program leaders said they would recommend the program to future leaders. However, they reported challenges with implementation due to equipment availability and behavioral challenges. Results suggest a comprehensive physical literacy program during the afterschool period can be feasible to implement and can lead to improvements in the affective domain of children’s physical literacy. Further research on childhood physical literacy interventions is warranted.

## 1. Introduction

Physical inactivity is a global problem among children and youth [1]. In Canada, for example, only one-third of children are meeting daily physical activity recommendations, with just under one-fifth of children meeting all three of the current 24-h movement guidelines regarding physical activity, sleep, and screen time [2]. Further, there is an alarming gap between boys and girls with boys engaging in significantly more physical activity and meeting the 24-h movement guidelines at a significantly higher rate than girls [2]. This discrepancy in participation is troublesome given the numerous physical, mental, and cognitive health benefits of physical activity for children and youth [3,4,5]. As such, strategies to increase the physical activity levels of all children and youth continue to be a global priority, with a growing emphasis on increasing the participation of girls and women [6]. Yet, despite ongoing efforts to increase physical activity levels, most physical activity interventions are largely unsuccessful at sustaining any long-term gains [7,8,9,10,11].

Although the school setting is often seen as an ideal environment to increase physical activity levels [12], most evidence suggests that school-based physical activity interventions have a limited impact on activity levels outside of the school day [13]. The afterschool period is therefore another time of day that may provide an opportunity for intervention. As such, a number of municipalities and sport and recreation organizations have worked to implement physical activity-based afterschool programming. However, much like school-day initiatives, the evidence regarding the efficacy of afterschool physical activity interventions is limited [14,15]. This may be due in part to implementation challenges experienced in the afterschool setting, insufficient training for afterschool program leaders, the program content not being sufficient to elicit a change in activity levels; or some combination of one or more of these challenges.

Regardless of setting, it is critical that any intervention be grounded in a theory (or model) of behavior change [16]. Indeed, several psychological theories have often been applied to understanding physical activity behavior [17,18,19,20] and more recently, researchers have proposed models that incorporate several psychological theories. Although many physical activity interventions have been developed based on psychological theories of behavior change, the effects of these interventions remain relatively small [21,22,23]. Similarly, interventions grounded in models of motor development [24,25] have also shown relatively small positive effects on physical activity behavior [26]. Thus, future interventions targeting physical activity may benefit from incorporating theories (or models) from several disciplines, such as across sport/exercise psychology and motor development, in order to target various physical (i.e., motor competence) and psychological (i.e., motivation) constructs altogether.

One potential solution to address the issue of theories or models spanning multiple disciplines regarding physical activity participation is employing an intervention grounded in physical literacy. Physical literacy is a multidimensional concept that includes the domains of movement competence, positive affect, confidence, and motivation necessary for regular engagement in physical activity [27]. Physical literacy, in a sense, unifies previous theories and models from sport/exercise psychology and motor development. Conceptually, physical literacy is linked to improved physical, mental, and social health through participation in physical activity [28]. However, physical literacy also provides a theoretical framework by which to guide intervention design. The importance of physical literacy is highlighted by its inclusion in national and international policies including the Physical Activity Action Plan for Canada and the World Health Organization’s Global Action Plan on Physical Activity [29,30]. Given the increasing emphasis on physical literacy, a number of stakeholders have focused their efforts on improving physical literacy in school-aged children. Yet, there are few evidence-based examples (e.g., [31,32,33]) of how to intentionally target physical literacy as a whole, and the effect this will have on physical literacy and physical activity levels.

Although a strength of physical literacy is the synthesis of psychological constructs combined with motor competence, we must also consider how other critical factors not included in the concept may influence or are influenced by physical literacy, and how they impact physical activity. One concept that is critical is gender. In this context, we use gender over sex given that the latter is used to refer to biological differences between males and females whereas the former recognizes how sex-based differences can be influenced by social and psychological factors [34]. In childhood and adolescence, there are well-known gender differences in physical activity with boys engaging in physical activity at a higher rate than girls [2]. Moreover, girls tend to drop-out of sport and physical activity at a higher rate than boys [35,36]. These discrepancies in physical activity may be due to many factors including societal norms about girls’ participation in physical activity [36], as well as differences in aspects of physical literacy including movement skill competence, confidence, enjoyment, and motivation to be active [37,38,39]. Thus, it is critical that we consider the role of gender in the design and analysis of physical literacy interventions.

The purpose of this study was to design and evaluate a physical literacy intervention for the afterschool setting. Specifically, we examined the effect of the intervention on the components of physical literacy in 7–13-year-old children and youth. We also examined the feasibility and acceptability of program implementation through evaluations of the leader training as well as through leader feedback following program implementation.

## 2. Materials and Methods

### 2.1. Participants and Design

Our target sample size was 90 afterschool participants, 7–13 years of age, based on a sample size calculation of the change in movement competence using a moderate effect (Cohen’s *d* = 0.60; [40]), an alpha of 0.05, and 80% power. In order to generate a sample of 90 participants we decided to randomly sample by program from the 386 afterschool programs in Ontario. Logistically, however, we did not have the research funds to travel to locations across the province. Therefore, we restricted the program sampling frame to programs located in the greater Hamilton region, a catchment area within a 30-min car drive of our lab and university. We initially contacted 16 sites, 12 of which were located within the City of Hamilton. Our initial enrolment target was a minimum of 15 children enrolled in the study from each site; however, this target was difficult to meet at all sites. As a result, we lowered our enrollment target to six participants per site to allow us to meet the overall target sample size of 90. Of the 16 sites contacted, two declined and one did not respond to our invitation to participate. An additional five sites did not reach our minimum enrolment targets. We therefore had eight sites who agreed to participate in the intervention and met enrolment targets; each of these sites were located within the City of Hamilton. These eight sites were then randomized to participate in either the experimental (*N* = 4) or control (*N* = 4) group. Participants in the experimental sites engaged in a 12-week physical literacy intervention, implemented by the site’s afterschool program leader. Participants in the control sites engaged in their usual afterschool program activities for the 12 weeks.

All children participating in the afterschool programs across the eight sites were approached by members of the research team to participate in the study. We received written parental consent and child assent from 96 children. All program leaders (*n* = 7) at the participating experimental sites (*N* = 4) also provided written informed consent to participate in the study. Parents of all participating children provided informed written consent and participants provided written child assent, while all program leaders provided informed written consent prior to participation in the study. The study was conducted in accordance with the Declaration of Helsinki, and the protocol was approved by the Hamilton Integrated Research Ethics Board (Project #1182).

### 2.2. Procedure

Child-level outcomes were measured using direct assessments of movement competence and a questionnaire administered at two points: baseline (prior to the commencement of the intervention) and post-intervention. Program leader outcomes were measured using a questionnaire administered at three time points: Pre- and post-leader training and post-intervention.

### 2.3. Intervention

The intervention was designed to deliberately target each domain of physical literacy through the inclusion of multiple teaching and intervention strategies. The movement competence component of the intervention was primarily based upon a previous 12-week curriculum designed by Ontario Physical and Health Education Association (OPHEA) and Sport for Life (https://sportforlife.ca) using OPHEA’s PlaySport activities (http://www.playsport.net/). The intervention was composed of two main components: skill stations and an active game. The program was designed to run 30 min per day, five days per week, for 12-weeks. The 12 weeks were divided into 20 skill blocks, with each block lasting three days. Each of these skill blocks focused on learning and practicing a different set of fundamental movement skills (e.g., jumping, throwing, catching) during the first 15 min of the program, with the remaining 15 min consisting of a novel active game incorporating the day’s movements. All active games were chosen from the PlaySport activities. The level of difficulty of both the skill stations and active game progressed in difficulty over the course of the three-day skill block and more generally over the 12-week intervention. This intervention was designed using a teaching games for understanding approach [41] and the inclusion of skill blocks and active games were chosen to target multiple aspects of the physical literacy cycle. Such an approach has been identified as consistent with a physical literacy-based approach [41] in that it emphasizes the importance of using novel games to learn new skills, the application of those skills in group-based settings, and how breaking down games into its skill components demonstrates the transferability of the skills that underlie sport and physical activity. Our inclusion of skill blocks also used a mastery approach whereby appropriate challenges were set, allowing children to experience the competence-confidence cycle emphasized in the physical literacy model [42]. The mastery climate also reinforced personal growth and the activities were designed to be challenging, yet scalable to ability, and fun. In all, the design of the intervention sought to create an experiential convergence of motor, affective, and social elements as described by Cairney et al. [28].

In addition, the curriculum targeted motivation and confidence (or self-efficacy) through several approaches. A general culture of mentoring and support was established within the program using two main strategies focusing on inclusivity and peer-modelling and support. Inclusivity referred to the creation of both a gender- and skill-inclusive environment that focused on ensuring all children had the opportunity to learn and practice new skills, regardless of their gender or level of movement skill proficiency. This culture of inclusivity was created through the leader training, a child workshop which set the tone for the intervention, and weekly tips sent to program leaders. In addition, the importance of peer modelling and support was discussed during the leader training and child workshops as a means for improving children’s confidence and, ultimately, motivation for engaging in sports and physical activity.

During both the skill station and active game portions of the intervention, pairings and groups were made to be inclusive of both gender and skill. Confidence and motivation were targeted through strategies aimed at increasing Bandura’s (1997) four sources of self-efficacy: Past performance mastery, vicarious experiences, verbal persuasion, and physiological/affective states. However, as children and youth often struggle to gauge their own self-efficacy [43], they often rely on both verbal and nonverbal cues from influential others (i.e., peers, leaders, and coaches) to inform their efficacy beliefs. Therefore, based on Lent and Lopez’s [44] extension of Bandura’s [45] theory of self-efficacy, two other forms of efficacy perceptions (i.e., other-efficacy and relation-inferred self-efficacy) that are informed in relational contexts and, in turn, influence one’s self-efficacy were targeted. *Other-efficacy* refers to one’s beliefs in another’s ability. For example, in an afterschool setting, a child could have other-efficacy perceptions about a program leader’s ability to teach them how to kick a ball. *Relation-inferred self-efficacy (RISE)* refers to one’s estimate about another’s belief in one’s ability. For example, this would refer to a child’s estimate of their coach’s belief in their own ability to kick a ball. In other words, if the child thinks the coach believes in their ability to kick a ball, then their own self-efficacy for kicking a ball would be enhanced. Indeed, children often report seeking both verbal and nonverbal cues from influential others in sport or exercise settings to inform their RISE and self-efficacy beliefs [46,47,48].

In order to enhance self-efficacy, other efficacy and RISE, modelling and support (from leaders and peers) were used extensively in all aspects of the intervention. For instance, leaders first demonstrated the skills and then children completed the skill stations in pairs whereby children worked together to teach each other how to perform the skill. Additionally, children were encouraged to model behavior and skills to one another during the active games. Based on previous research [47], coaches and children were also encouraged to provide RISE-enhancing verbal feedback (e.g., “*I believe in you*”, “*I’m confident you are going to do really well*”) to children when they were practicing the skills and during the active games. All program leaders were provided with information during training (see below) and in the manual on how to use RISE-enhancing cues in order to increase the self-efficacy of the children.

### 2.4. Program Leader Training

Afterschool Program Leaders from the experimental sites attended a 2-h training session prior to the start of the program. Consistent with previous research [48,49] and social cognitive theory [50], the training program consisted of two-phases designed to enhance the leaders’ perceived knowledge, outcome expectations, self-efficacy, and intentions for delivering a physical literacy-based intervention. The first phase of the training consisted of a classroom-based workshop that included presenting information on the importance of physical literacy, the status of physical literacy levels of Canadian children and youth, the need to create a gender- and skill-inclusive environment, and the importance of instilling confidence in children through RISE-enhancing feedback. The second phase involved an experiential learning component whereby program leaders were then shown how to use the manual and supporting documents and were led through an interactive hands-on practice session. Program leaders in the experimental group (*N* = 7) were also emailed a weekly tip on how to make their program more gender inclusive. They also received a weekly call for the first three weeks of the program, during which a trained member of the research team used techniques from motivational interviewing [51] to help the leaders create Brief Action Plans for the upcoming week [52] of their program in order to meet their goals for program implementation as well as deal with any issues that arose during the previous week.

### 2.5. Measures

Given that physical literacy is a multi-dimensional concept, we employed a measurement strategy to capture each of the core domains, consistent with the approach advocated by Barnett et al. [53].

#### 2.5.1. Physical Literacy—Movement Competence Domain

Movement competence of participating children was assessed at each time point with the PLAYfun tool [54]. PLAYfun consists of 18 movement tasks, completed by participants, and scored by trained assessors using a modified 100 mm visual analogue scale. The scores on the 18 tasks are averaged to create an average PLAYfun score, as well as individual domain scores across five respective domains: running, locomotor, object control—upper body, object control—lower body, and balance. Complete scoring criteria for each of the tasks can be found in the manual (https://play.physicalliteracy.ca/play-tools/playfun). The PLAYfun tool has previously been shown to have acceptable construct validity through both confirmatory factor analysis [55] and a convergent validation study [56]. All assessors underwent substantial training on the tool, had excellent inter-rater reliability (>90%), and were blind to group assignment.

#### 2.5.2. Physical Literacy—Cognitive and Affective Domains

Children completed a 92-item questionnaire to assess demographic variables (at baseline only), their self-efficacy, other-efficacy (i.e., their belief in their leaders and peers’ abilities), RISE (i.e., their belief about what they think their leaders and peers think about them), and motivation and enjoyment to engage in physical activity pre- and post-intervention (see below). This questionnaire was based on previous work completed by our lab and consisted of task appropriate questions created specifically for this study, in addition to the PLAYself survey [54]. The items assessing self-efficacy, other-efficacy, and RISE were developed by adhering to Bandura’s [57] recommendations for constructing self-efficacy scales and were based on previous research that assessed these constructs in youth [46,58]. Motivation and enjoyment were assessed using items from the Intrinsic Motivation Inventory [59], which has been validated for use in sport and exercise settings [60] and has been successfully used with children [61].

##### Demographic Information

Children reported their date of birth, gender, ethnicity/cultural heritage, and the composition of their household.

##### Self-Efficacy

Adhering to recommendations by Bandura [57], self-efficacy was assessed using a 16-item scale. Each item was prefaced with the stem *“How confident are you in your ability”*. The individual items represented various perceptions in relation to when the child is engaging in sports and physical activity at their afterschool program. Children rated their confidence for each item using an 11-point scale from 0 (*not confident*) to 10 (*totally confident*). An example item is: “*To engage in physical activity (e.g., running, biking)*”. The self-efficacy score was computed by averaging the ratings for each item to produce a scale value out of 10. The average internal consistency of the scale across the two time points was good (Cronbach’s α = 0.84).

##### Other-Efficacy—Leader

Children’s other-efficacy perceptions with regards to their primary group leader was assessed using 8-items. Each item was prefaced with the stem “*How confident are you in your group leaders’ ability*”. The individual items represented the child’s belief in their group leaders’ ability to provide guidance and encouragement with regards to when the child is engaging in sports and physical activity at their afterschool program. Children rated their confidence for each item using an 11-point scale from 0 (not confident) to 10 (totally confident). An example item is: “*To teach you the skills to play the sports and games*”. The other-efficacy—leader score was computed by averaging the ratings for each item to produce a scale value out of 10. The average internal consistency of the scale across the two time points was good (α = 0.87).

##### Other-Efficacy—Peer

Similar to the above, children’s other-efficacy perceptions with regards to their peers was assessed using the same 8-items. However, each item was prefaced with the stem *“How confident are you in your friends’ and peers’ ability”*. The individual items represented the child’s belief in their friends’ and peers’ abilities to provide guidance and encouragement with regards to when the child is engaging in sports and physical activity at their afterschool program. Children rated their confidence for each item using an 11-point scale from 0 (*not confident*) to 10 (*totally confident*). An example item is: “*To encourage you, even when you make a mistake or find something difficult*”. The other-efficacy—peer score was computed by averaging the ratings for each item to produce a scale value out of 10. The average internal consistency of the scale across the two time points was good (α = 0.88).

##### Relation-Inferred Self-Efficacy—Leader

Children’s RISE perceptions with regards to their primary group leader was assessed using 12-items. Each item was prefaced with the stem *“How confident do you think your group leader is in your ability”*. The individual items represented the child’s belief in their group leader’s belief in their own abilities in relation to when the child is engaging in sports and physical activity at their afterschool program. Children rated their confidence for each item using an 11-point scale from 0 (*not confident*) to 10 (*totally confident*). An example item is: “*To engage in sports (e.g., baseball, soccer)*”. The RISE—leader score was computed by averaging the ratings for each item to produce a scale value out of 10. The average internal consistency of the scale across the two time points was excellent (α = 0.91).

##### Relation-Inferred Self-Efficacy—Peer

Similar to the above, children’s RISE perceptions with regards to their friends and peers was assessed using 12-items. Each item was prefaced with the stem *“How confident do you think your friends and peers are in your ability”*. The individual items represented the child’s belief in their friends and peers’ beliefs in their own abilities in relation to when the child is engaging in sports and physical activity at their afterschool program. Children rated their confidence for each item using an 11-point scale from 0 (*not confident*) to 10 (*totally confident*). An example item is: “*To jump, hop, and skip*”. The RISE—peer score was computed by averaging the ratings for each item to produce a scale value out of 10. The average internal consistency of the scale across the two time points was excellent (α = 0.91).

##### Motivation

Motivation for engaging in physical activity and sports within the afterschool setting was assessed using the effort and importance subscale from the Intrinsic Motivation Inventory [59]. The effort and importance subscale is a five-item seven-point Likert-type scale ranging from 1 (*not at all true)* to 7 (*very true*). Each item was prefaced with the following stem “*For each of the following statements, please indicate how true it is for you when you are engaging in physical activity and sports at your afterschool program*”. An example item is: “*I put a lot of effort into sports and games*”. The motivation score was computed by averaging the ratings for each item to produce a scale value out of seven. The average internal consistency of the scale across the two time points was good (α = 0.84).

##### Enjoyment

Enjoyment for engaging in physical and sports within the afterschool setting was assessed using 2-items from the interest/enjoyment subscale from the Intrinsic Motivation Inventory (Ryan, 1982) rated on a seven-point Likert-type scale ranging from 1 (*not at all true)* to 7 (*very true*). Each item was prefaced with the following stem “*For each of the following statements, please indicate how true it is for you when you are engaging in physical activity and sports at your afterschool program”*. The two-items include “*I enjoy playing sports and games*” and “*I have fun playing sports and games*”. The enjoyment score was computed by averaging the ratings for each item to produce a scale value out of seven. The average internal consistency of the scale across the two time points was good (α = 0.85).

##### PLAYself

The PLAYself survey was completed by participants as a self-evaluation of their perception of their physical literacy [54] (https://play.physicalliteracy.ca/play-tools/playself). PLAYself includes 21-items, across four domains including perceptions of the participant’s confidence in different environments (e.g., water, snow, playground); their self-efficacy toward engaging in physical activity; and their relative ranking of different literacies (i.e., literacy, numeracy, physical literacy). The PLAYself has demonstrated excellent test-retest reliability (0.83) in children 6–14 years of age, along with convergent validity consistent with definitions of physical literacy (P. Jefferies, personal communication, April 2020). The average internal consistency of the measure across the two time points was good (α = 0.84).

#### 2.5.3. Program Leader Questionnaire

Based on previous research [48] and constructs contained within social cognitive theory [62,63] program leaders completed a 54-item questionnaire pre- and post-training, in addition to post-intervention, in order to assess social cognitive variables (i.e., perceived knowledge, outcome expectations, self-efficacy, and intentions) toward implementing aspects of the physical literacy intervention (e.g., knowledge of ways to provide verbal and non-verbal feedback to increase RISE, knowledge of ways to create gender and skill-inclusive learning and playing environments). The post-intervention questionnaire also included open-ended questions to elicit the program leaders’ feedback on the program. As we were interested in program leaders’ state perceptions, and whether these changed over time, program leaders were reminded to report on their beliefs “at this point in time”. The questions included in the program leader questionnaire can be found in Table S1. Items from this questionnaire have been used successfully in past research [48] when assessing perceived knowledge, outcome expectations, self-efficacy, and intentions before and after a RISE workshop including young adult and adult participants, and were created adhering to recommendations ([58]; also see [59]).

##### Perceived Knowledge

Program leaders’ perceived knowledge regarding methods for providing RISE-enhancing information, and for creating gender and skill inclusive learning and playing environments was assessed with 10-items developed for this study. Each item was prefaced with the following stem “*To what extent are you knowledgeable about*” and rated on a 7-point Likert-type scale ranging from 1 (*not very knowledgeable)* to 7 (*very knowledgeable*). Example items include “*The best things to say in order to effectively communicate your confidence in your students’ abilities*” and “*The best ways to create physical activity and sport learning environments consisting of higher skilled and lower skilled children*”. The perceived knowledge score was computed by averaging the ratings for each item to produce a scale value out of 7. The average internal consistency of the measure across the three time points was acceptable (α = 0.76).

##### Outcome Expectations

Program leaders’ beliefs about the usefulness of providing RISE-enhancing information, and for creating gender and skill inclusive learning and playing environments was assessed with 15-items developed for this study. Each item was rated on a 7-point Likert-type scale ranging from 1 (*strongly disagree*) to 7 (*strongly agree*). Example items include “*To what extent do you think providing non-verbal feedback helps kids develop confidence in their physical activity and sport abilities*”, *“To what extent do you think it is important to create gender inclusive physical activity and sport playing environments”,* “*Effectively communicating confidence in my students’ abilities would make them feel more confident in their own abilities*”, and “*Effectively communicating confidence in my students’ abilities would motivate them to attempt things they haven’t done before*”. The outcome expectations score was computed by averaging the ratings for each item to produce a scale value out of 7. The average internal consistency of the measure across the three time points was excellent (α = 0.93).

##### Self-Efficacy

Program leaders’ beliefs in their abilities for providing RISE-enhancing information, and for creating gender and skill inclusive learning and playing environments was assessed with 11-items developed for this study. Each item was prefaced with the following stem “*How confident are you in your ability to*” and rated on a 7-point Likert-type scale ranging from 1 (*not confidence at all)* to 7 (*completely confident*). Example items include “*Use verbal feedback to effectively communicate your belief in your students’ abilities*” and “*Identify appropriate situations for which to communicate your belief in your students’ abilities*”. The self-efficacy score was computed by averaging the ratings for each item to produce a scale value out of 7. The average internal consistency of the measure across the three time points was excellent (α = 0.92).

##### Intentions

Program leaders’ intentions for providing RISE-enhancing information, and for creating gender and skill inclusive learning and playing environments was assessed at two time points (post-training and post-intervention) with 9-items developed for this study. Each item was rated on a 7-point Likert-type scale ranging from 1 (*not true at all*) to 7 (*very true*). Example items include “*I intend to use non-verbal feedback to effectively communicate your belief in your students’ abilities*” and “*I intend on communicating my confidence in my students’ physical and sport abilities during every physical activity session*”. The intention score was computed by averaging the ratings for each item to produce a scale value out of 7. The average internal consistency of the measure across the two time points was good (α = 0.83).

##### Program Feedback

Program leaders were asked five open-ended questions post-intervention to solicit their feedback on the program. These questions asked (1) whether they would recommend the program; (2) if there was anything they would like to change about the program; (3) their favorite part of the program; (4) their least favorite part of the program; and (5) any additional feedback or comments about the program.

### 2.6. Analysis

Descriptive statistics were calculated for child and leader demographic and outcome variables. Independent samples t-tests were used to examine differences between the experimental and control groups on age and all outcome variables at baseline. Chi-squared tests were used to examine differences between the groups on all other demographic variables at baseline. Three repeated measures ANOVAs were used to assess change in the program leaders’ outcome expectations, perceived knowledge, and self-efficacy across the three time points (pre-training, post-training, post-intervention). A paired samples t-test was used to assess change in the program leaders’ intentions from post-training to post-intervention. Effect sizes for the ANOVAs are reported as partial eta squared (η_p_^2^) and the values for small, medium, and large effects are 0.01, 0.06, and 0.14, respectively [64]. Effect sizes for the t-tests are reported as Cohen’s *d* and the values for small, medium, and large effects are 0.2, 0.5, and 0.8, respectively [64]. A series of nine linear regression models were conducted to examine the effect of the intervention (change in each of the nine child-level outcomes from baseline to post-intervention for both the control and experimental groups), controlling for age, gender, baseline score on the outcome of interest, and adjusting for clustering within sites. Assumptions for multicollinearity and autocorrelation were met in each of the nine models with the VIFs of each predictor ranging from 1–3, and the Durbin Watson test statistics ranging from 1.5–2.5, respectively [65]. All quantitative analyses were conducted in SPSS 25 [66].

Finally, open-ended survey responses from the program leaders, regarding their feedback on the program, are provided verbatim to add context on the feasibility and acceptability of the program.

## 3. Results

### 3.1. Program Leader Outcomes

Seven program leaders took part in the study. These leaders were mostly female and had, on average, two years of experience leading afterschool programs. Demographic characteristics of the program leaders are presented in Table 1.

Changes in the program leaders’ outcome expectations, perceived knowledge, self-efficacy, and intentions are presented in Table 2 and indicate that the training generally had a positive impact on these outcomes. Specifically, their perceived knowledge and self-efficacy to implement a physical literacy program significantly increased from pre- to post-training and these improvements were maintained at post-intervention.

### 3.2. Child Outcomes

A total of 96 children provided consent for the study; however, six of these children were absent for baseline testing. Therefore, the final sample included 90 children. Of these, 47 received the intervention, while 43 served as controls. The post-tests were completed by 38 participants in the experimental group and 28 participants in the control group with attrition due to children no longer attending their respective afterschool program. The demographic characteristics of the sample at baseline are shown in Table 3. Only age was significant, with children in the control group being slightly older than children in the experimental group. As sites were randomized to condition, differences in age between arms of the study is due to chance. Table 4 presents baseline scores on all outcomes, by condition. The control group scored significantly better than the experimental group on the PLAYfun at baseline, possibly due to age differences. No other baseline differences were present between the groups.

There were no significant differences observed in the change of motor competence from baseline to post-intervention between the experimental and control groups, after adjustment for age, gender, baseline score, and site. However, baseline scores in motor competence did predict post-intervention motor competence; children who scored higher on PLAYfun at baseline tended to perform better post-intervention. As well, older children and boys also performed better on the post-assessment of motor competence. Similarly, baseline scores on the cognitive and affective domains (i.e., self-efficacy, motivation, enjoyment, PLAYself, other-efficacy, and RISE) predicted post-intervention scores on these outcomes, with those children who had higher scores at baseline performing better post-intervention. There were, however, significant differences observed in the change of enjoyment, other-efficacy—leader, and RISE—leader from baseline to post-intervention between the experimental and control groups, after adjustment for age, gender, baseline score, and site; children in the experimental group reported higher scores on these outcomes post-intervention. Intervention effects are reported in Table 5.

### 3.3. Program Feedback

Verbatim responses from the program leaders’ post-intervention feedback survey are presented in Table 6. Overall, the program leaders had positive feedback about the program although they did report some challenges with implementation. All program leaders reported that they would recommend the program to future program leaders; however, they had a few suggested changes to the program structure and administration. For example, two program leaders suggested that all equipment be supplied while other leaders suggested an increase in program length, the inclusion of free time, and an increased variety of activities. When asked about their least favorite part of the program, the leaders reported challenges with behavioral issues and keeping the participants engaged on a daily basis. Conversely, the leaders reported that their favorite part of the program was seeing children help one another and respond to positive feedback provided by their leaders. The leaders also reported that the children had fun participating in the novel games and that it was beneficial for the leaders to not have to prepare their own lesson plans.

## 4. Discussion

While there has been great interest in the concept of physical literacy at both the policy and programming level, empirical inquiry has lagged behind theoretical interest. Recently, researchers have begun to design and evaluate interventions that employ a physical literacy-based approach [31,32,33]. Yet, much of this work includes program evaluations of existing programs or interventions only targeting some of the domains of physical literacy. Further, none of these studies have taken place in the afterschool setting—a setting that has been identified as a critical target for physical activity programming [14]. To the best of our knowledge, this is the first study to empirically test a comprehensive physical literacy intervention in the afterschool setting.

Despite the comprehensive design of our physical literacy intervention, intervention effects were only evident in three of our child-level outcomes. However, the fact that we found a significant intervention effect on participants’ enjoyment, their efficacy in their leaders, and their perceived RISE from their leaders is quite promising. These findings suggest that participating in the intervention led to increased enjoyment in physical activity and sports. Further, it demonstrates that participants gained confidence in their group leader’s ability to teach them and increased their perception of their group leader’s confidence in their own abilities. These cognitive and affective outcomes are critical components of physical literacy and are necessary for regular participation in physical activity [28].

While the lack of an intervention effect on the remaining outcomes, particularly movement competence, is disappointing, it is not too surprising given the challenges of the afterschool space, as highlighted by the program leaders. Specifically, issues in securing equipment made implementation a challenge. Likewise, program leaders struggled with behavioral challenges, such as children acting out or not wanting to participate in afterschool activities, within the program. It is therefore unclear if the lack of intervention effect, across all variables, was simply due to low fidelity, rather than the intervention itself. These challenges in the afterschool setting are not unique to this study as previous research aimed at increasing physical activity through afterschool programming has also demonstrated mixed results [14]. It is unclear however, if the limited success of afterschool programs to increase physical activity is due to the afterschool setting, the intervention itself, or the general difficulty in increasing physical activity levels in school-aged children [7]. In contrast, previous research has demonstrated the effectiveness of improving movement competence in children and youth through school-based interventions [40,67]; however, this work has not focused specifically on the afterschool setting. More research is needed not only to examine the effectiveness of afterschool programs at improving physical literacy, but measures of feasibility and intervention fidelity in the afterschool setting.

Despite limited effects on the child-level outcomes, the results of the leader training are promising. That we were able to improve the cognitions of the afterschool program leaders through a brief two-hour training session suggests that the problem may not be with training the leaders but, with the afterschool setting itself. Indeed, we saw that the program leaders improved their perceived knowledge and self-efficacy for delivering a physical literacy-based afterschool intervention; and these improvements were maintained through the intervention period with minimal support. This is promising given that the program leaders had little to no prior knowledge of physical literacy, or how to implement a physical literacy program in the afterschool setting. It is possible, then, that with more comprehensive training we may be able to engage afterschool program leaders to not only deliver physical literacy programs but, also be better prepared to address some of the challenges associated with program implementation.

Despite the challenges of the afterschool setting for this type of intervention work, it is important that we continue to explore the role of comprehensive physical literacy interventions for school-aged children and youth. However, it may be more optimal to test these effects in a more controlled environment, such as during the school day. This could potentially limit barriers to implementation that were faced in the present study including limited access to the gymnasium and children being picked-up early from afterschool programming. Kriellaars and colleagues [31], for instance, recently evaluated a physical literacy-inspired circus arts instruction program for the physical education setting. While they found that both traditional physical education and physical literacy-inspired circus arts instruction led to significant improvements in movement competence, endpoint scores in movement competence favored the physical literacy-inspired circus arts [31]. These results suggest that physical literacy programming, delivered during the school day, may be an effective way to improve aspects of children’s physical literacy beyond gains seen in traditional physical education. Yet, in addition to testing physical literacy programs in more controlled settings, such as in school, it is important that we continue to test how we can support afterschool program leaders in the implementation of physical literacy programs. This may mean enhanced training for the program leaders and on-going support over the duration of the intervention.

Limitations include the relatively small sample size given the multiple sites included in the study. Further, the included sites were from a geographically narrow region. We were also limited by our ability to control intervention fidelity and the variable environment of the afterschool setting. While a qualitative analysis, using a thematic or content analysis approach, may have provided further insight into the survey responses [68,69] this was beyond the scope of this study, which may have limited our ability to gain an even deeper understanding of the program leaders’ post-program feedback. However, the leaders’ responses did provide insight into some of the challenges they experienced with program implementation. For example, feedback from the program leaders revealed that each site was unique in its ability to access resources such as space and equipment. Many sites indicated that this access was limited (e.g., only having access to the gymnasium two or three days per week, rather than all five days) and negatively affected program implementation. Further, despite randomization, it appeared that some of the control sites had more regular access to the gymnasium and equipment than the experimental sites. That participants in the control group may have had greater opportunities to be active could account for some of our null findings. It is possible that additional, unmeasured, variables may also help to account for these findings as we were unable to measure individual- or school-level socioeconomic variables such as parental education, income, and other variables that may have differed between the sites. We recommend that future research include measures of additional individual- and site-level variables that could influence program outcomes. A final limitation is that the results of the program leaders’ cognitions may be biased given the potential social desirability of the leaders to respond positively to the training. Further, while we saw positive changes in their beliefs and intentions, these alone are often insufficient to actually change behavior [70,71]. Future work should observe the program leaders in practice to assess the impact of the training on their ability to implement the intervention. A strength of this study is the novelty of using a physical literacy-based framework to comprehensively intervene on and measure the core domains of physical literacy. While physical literacy is a holistic concept, it is important to consider and measure its component domains using valid assessments and a combination of direct and self-reported assessments.

## 5. Conclusions

In conclusion, it appears that a comprehensive 12-week physical literacy program implemented during the afterschool period can be feasible for non-experts to deliver and in the current study showed improvements in cognitive and affective domains of children’s physical literacy. More research is needed regarding the effect of comprehensive physical literacy programming on children’s physical literacy, and how this relates to their physical activity and associated health outcomes.

## Figures and Tables

**Table 1:** Descriptive characteristics of the program leaders at baseline.

	Mean or Frequency
Age (mean (SD))	31.1 (14.4)
Sex	
Female (*n*)	*6*
Male (*n*)	*1*
Race/Ethnicity	
White (*n*)	*3*
Black (*n*)	*2*
Other (*n*)	*2*
Level of Education	
Completed high school (*n*)	*1*
Some college or technical training (*n*)	*1*
Completed college or technical training (*n*)	*3*
Completed a bachelor’s degree (*n*)	*2*
Years of Experience as Program Leader	
<1 year (*n*)	*2*
1–2 years (*n*)	*3*
>2 years (*n*)	*2*
Previous Physical Activity Training	
No (*n*)	*3*
Yes (*n*)	*4*

**Table 2:** Change in program leaders’ outcomes across time.

Outcome	Pre-Training	Post-Training	Post-Intervention	Difference
Outcome expectations	6.4 (0.5)	6.5 (0.4)	6.6 (0.3)	F (2,12) = 1.22, *p* = 0.33, η_p_^2^ = 0.17
Perceived knowledge	4.8 (1.3)	6.2 (0.6) *	6.2 (0.6) *	F (2,12) = 7.41, *p* = 0.01, η_p_^2^ = 0.55
Self-efficacy	5.3 (0.7)	6.3 (0.5) *	6.4 (0.6) **	F (2,12) = 11.75, *p* = 0.001, η_p_^2^ = 0.66
Intentions	N/A	6.8 (0.4)	6.4 (0.7)	*t* (6) = 1.8, *p* = 0.13, *d* = 0.52

* Significantly different from pre-training (*p* < 0.05) ** Significantly different from pre-training (*p* < 0.01).

**Table 3:** Demographic characteristics of the participating children at baseline.

Variable	Experimental	Control	Difference
Age (mean (SD))	9.1 (1.4)	10.5 (1.8)	*t* = 4.1, *df* = 88, *p* < 0.001
Gender			
Girls (*n*)	23	19	*x*^2^ (1) = 0.20, *p* = 0.65
Boys (*n*)	24	24	
Race/ethnicity			
White (*n*)	32	33	*x*^2^ (1) = 0.58, *p* = 0.45
Other (*n*)	14	10	
Living situation			
Lives in one-home (*n*)	33	29	*x*^2^ (1) = 0.19, *p* = 0.66
Splits time between two homes (*n*)	13	14	
Parental place of birth			
Both parents born in Canada (*n*)	32	27	*x*^2^ (1) = 0.28, *p* = 0.60
One or both parents born elsewhere (*n*)	15	16	

**Table 4:** Baseline scores, by group, on all child-level outcomes.

Outcome	Experimental Mean (SD)	Control Mean (SD)	Effect Size (Cohen’s *d*)
PLAYfun average score	39.2 (9.8)	47.3 (8.9) *	0.86
Self-efficacy	7.6 (1.9)	7.5 (1.4)	0.06
Motivation	6.0 (1.4)	6.0 (1.2)	<0.01
Enjoyment	6.4 (1.2)	6.2 (1.4)	0.15
PLAYself total score	76.2 (13.4)	75.5 (13.0)	0.05
Other-efficacy—leader	8.2 (1.9)	7.9 (1.9)	0.16
Other-efficacy—peer	7.7 (2.3)	7.4 (2.1)	0.14
Relation-inferred self-efficacy (RISE)—leader	8.2 (2.2)	7.8 (1.8)	0.20
RISE—peer	7.8 (1.9)	7.6 (1.7)	0.11

Note: * significantly different from the experimental group at *p* < 0.001.

**Table 5:** Intervention effects on motor competence, cognitive, and affective outcomes.

Outcome	b (SE)	*p*-Value	r-Squared
**PLAYfun average score**			
Experimental group	−3.99 (2.36)	0.10	
Age (years)	1.52 (0.59)	0.01 **	
Boys	4.27 (1.48)	<0.01 **	
Baseline score	0.43 (0.10)	<0.001 ***	0.728
**Self-efficacy**			
Experimental group	0.07 (0.38)	0.85	
Age (years)	0.04 (0.09)	0.68	
Boys	0.03 (0.29)	0.93	
Baseline score	0.53 (0.08)	<0.001 ***	0.541
**Motivation**			
Experimental group	0.56 (0.38)	0.14	
Age (years)	−0.04 (0.09)	0.63	
Boys	0.38 (0.27)	0.17	
Baseline score	0.30 (0.11)	0.01 **	0.330
**Enjoyment**			
Experimental group	0.95 (0.43)	0.03 *	
Age (years)	−0.02 (0.10)	0.82	
Boys	0.38 (0.29)	0.20	
Baseline score	0.61 (0.14)	<0.001 ***	0.391
**PLAYself total score**			
Experimental group	0.61 (4.78)	0.90	
Age (years)	−0.17 (1.10)	0.88	
Boys	1.86 (3.25)	0.57	
Baseline score	0.36 (0.12)	<0.01 **	0.289
**Other-efficacy—Leader**			
Experimental group	2.92 (0.66)	<0.001 ***	
Age (years)	−0.04 (0.15)	0.76	
Boys	0.22 (0.43)	0.62	
Baseline score	0.41 (0.13)	<0.01 **	0.497
**Other-efficacy—Peer**			
Experimental group	0.07 (0.81)	0.93	
Age (years)	0.02 (0.18)	0.90	
Boys	1.18 (0.50)	0.02 *	
Baseline score	0.19 (0.11)	0.10	0.192
**RISE—Leader**			
Experimental group	1.14 (0.56)	0.05 *	
Age (years)	−0.07 (0.13)	0.59	
Boys	0.24 (0.37)	0.52	
Baseline score	0.55 (0.10)	<0.001 ***	0.494
**RISE—Peer**			
Experimental group	1.36 (0.81)	0.10	
Age (years)	−0.10 (0.19)	0.62	
Boys	0.85 (0.56)	0.13	
Baseline score	0.41 (0.17)	0.02 *	0.272

Note: * significant at *p* < 0.05; ** significant at *p* < 0.01; *** significant at *p* < 0.001.

**Table 6:** Post-intervention feedback from the program leaders.

Question	Program Leader Responses
Would you recommend the program to other program leaders? Please elaborate.	1. I would recommend the program to other leaders as long as they are not implementing another program and have participants who are used to structured programming. 2. Yes, this has increased our children’s participation in fundamental skills that they do not usually enjoy. 3. I would for programs that have a lot of structure. On the other hand, programs like mine are very hard to implement this. 4. Yes. It covers a wide range of physical activities to enhance PL skills. They are clearly explained and offer a variety of games with and without equipment. It encourages children to lead, as well in terms of skill sharing. 5. I would recommend to other program leaders because it is a great way of keeping a group of children occupied and entertained. This program has taught me a number of different gym games that cover a variety of categories. 6. Yes! The lesson plans were easy to follow so long as you have the right kind (or any) equipment. 7. Yes, because it helps build the confidence in the children and helps leaders to learn new gym games.
Is there anything you would like to change about the program? Please describe.	1. Supply all equipment and supplies at the beginning of the program to leaders who are implementing the program. 2. An increase in the variety of activities would be helpful in keeping children engaged. 3. Maybe have some free time. 4. Could the font size be a bit larger for text in booklet? 5. The only thing I would change were the games given as examples in the books. It became difficult to think of certain activities. 6. 12 Weeks is (in my opinion) too short to make true lasting attitude change. I think also 3-day blocks are too short. 7. The thing I would like to change is taking into consideration that we didn’t have the proper equipment.
What was your favorite part about the program?	1. Introducing new skills to some participants while helping others maintain and develop certain skills. 2. My favorite part of the program was seeing kids helping each other with peer-based learning. 3. Diverse games 4. Seeing children interact with enthusiasm when participating in whole group games or in pairs practicing their skills and also responding to positive feedback from their leaders. When a child developed a skill and recognized their own progress for those skills. 5. Seeing the kids having fun with the various games. 6. The new games—kids loved them. Not having to prepare plans was great. 7. Everything
What was your least favorite part about the program?	1. Overwhelmed due to implementing more than one program plan. 2. My least favorite part of the program was keeping children engaged in repeat games/activities and having to keep up engagement. 3. How short the weeks were. 4. Sometimes, children may not always want to have 3-day blocks and also some children due to behavioral issues, wanted to do their own thing (regardless of what was presented)—They are this way prior to the program. 5. Getting the kids to participate. 6. Having to meet deadlines and “strict” structure. I think structure is important though, and kids need structure, but it was too tight. 7. Not having the proper equipment for some games.
Do you have any additional feedback or comments about the program?	1. Staying in contact with staff throughout the duration of program was great support. Supply equipment at initial training in order for staff to properly implement activities and skill stations. 2. I think this was very helpful in increasing participant skills and increasing the idea of gender inclusivity (not all children fully understand this concept yet, but it is starting to set in). 3. N/A 4. Could an abridged version be offered with featuring a variety of activities but more economizing on use of words/explanations? 5. Hopefully this study sticks around in the future. 6. 5-day blocks. Rotation after 5 days of activity from block 1 to 6. 7. No.

## Supplementary Materials

The following are available online at https://www.mdpi.com/1660-4601/17/9/3129/s1, Table S1: Questions included in the program leader questionnaire.
