# Supplementary material for: Outcomes and Feasibility of a 12-Week Physical Literacy Intervention for Children in an Afterschool Program
Source: Int J Environ Res Public Health. 2020 Apr 30;17(9):3129. doi: 10.3390/ijerph17093129 (PMC7246927; doi:10.3390/ijerph17093129)
Supplement: Supplementary file 1 [file ijerph-17-03129-s001.pdf]

Supplementary Material

Table S1. Questions included in the program leader questionnaire.

Outcome	Question Stem	Question
Perceived Knowledge	To what extent are you knowledgeable about:	The best things to say in order to effectively communicate your confidence in your students' abilities?
		Best things to do in order to effectively communicate your confidence in your students' abilities?
		The best times/situations to effectively communicate your confidence in your students' abilities during physical activity sessions?
		The best ways to effectively tailor your feedback to different students?
		The best ways to instruct peers to help kids develop confidence in each other's physical activity and sport abilities?
		The best ways to create gender inclusive physical activity and sport learning environments?
		The best ways to create gender inclusive physical activity and sport playing environments?
		The best ways to create physical activity and sport learning environments where higher skilled children instruct lower skilled children?
		The best ways to create mixed physical activity and sport playing environments consisting of higher skilled and lower skilled children?
		The [program] physical activity lesson plans?
Outcome Expectations	At this point in time:	To what extent do you think providing verbal feedback helps kids develop confidence in their physical activity and sport abilities?
		To what extent do you think providing non-verbal feedback helps kids develop confidence in their physical activity and sport abilities?
		To what extent do you think that peers can help kids develop confidence in each other's physical activity and sport abilities?
		To what extent do you think that males are better than females at physical activity and sport tasks?
		To what extent do you think it is important to create gender inclusive physical activity and sport learning environments?
		To what extent do you think it is important to create gender inclusive physical activity and sport playing environments?
		To what extent do you think that it is important to create physical activity and sport learning environments where higher skilled children instruct lower skilled children?
		To what extent do you think that it is important to create mixed physical activity and sport playing environments consisting of higher skilled and lower skilled children?
	Effectively communicating confidence in my students' abilities would:	Make them feel more confident in their own abilities.
		Motivate them to attempt things they haven't done before.

		Make them try harder when they attempt difficult tasks.
		Help them keep trying harder when they are practicing difficult skills.
		Make them feel less anxious about performing difficult tasks.
		Enhance the impact of my instructions (as a leader).
		Improve my relationship with them (as a leader).
Self-efficacy	How confident are you in your ability to?	Use verbal feedback to effectively communicate your belief in your students' abilities?
		Use non-verbal feedback to effectively communicate your belief in your students' abilities?
		Effectively communicate your belief in your students' abilities during every physical activity session?
		Communicate your belief in your students' abilities in a way that seems genuine?
		Identify appropriate situations for which to communicate your belief in your students' abilities?
		Instruct peers to help kids develop confidence in each other's physical activity and sport abilities?
		Create gender inclusive physical activity and sport learning environments?
		Create gender inclusive physical activity and sport playing environments?
		Create physical activity and sport learning environments where higher skilled children instruct lower skilled children?
		Create mixed learning and playing environments consisting of higher skilled and lower skilled children?
		Administer the [program] physical activity lesson plans?
Intentions (only measured post-training and post-intervention)	At this point in time:	I intend to use verbal feedback to effectively communicate my confidence in my students' physical activity and sport abilities.
		I intend to use non-verbal feedback to effectively communicate my confidence in my students' physical activity and sport abilities.
		I intend on communicating my confidence in my students' physical and sport abilities during every physical activity session.
		I intend to use peers to help kids develop confidence in each other's physical activity and sport abilities.
		I intend to use gender inclusive physical activity and sport learning environments.
		I intend to use gender inclusive physical activity and sport playing environments.
		I intend to use higher skilled children to instruct lower skilled children.
		I intend to use mixed playing environments consisting of higher and lower skilled children.
		I intend to use the [program] physical activity lesson plans each week.
